# The functional and molecular studies on involvement of hydrogen sulphide in myometrial activity of non-pregnant buffaloes (*Bubalus bubalis*)

**DOI:** 10.1186/s12917-017-1288-9

**Published:** 2017-12-06

**Authors:** Sooraj V. Nair, Vipin Sharma, Abhishek Sharma, Udayraj P. Nakade, Pooja Jaitley, Karikalan Mathesh, Soumen Choudhury, Satish Kumar Garg

**Affiliations:** 1 Experimental and Molecular Pharmacology Laboratory, Department of Pharmacology & Toxicology, College of Veterinary Science and Animal Husbandry, U.P. Pandit Deen Dayal Upadhyaya Pashu Chikitsa Vigyan Vishwavidyalaya Evam Go-Anusandhan Sansthan (DUVASU), Mathura, 281001 India; 2 Centre for Wildlife Conservation Management and Disease Surveillance, Indian Veterinary Research Institute (ICAR-IVRI), Izatnagar, Bareilly, U.P 243 122 India

**Keywords:** L-type Ca^2+^ channel, H_2_S, L-Cysteine, Non-pregnant buffalo, Myometrium

## Abstract

**Background:**

Hydrogen sulphide (H_2_S), a member of the gasotransmitters family, is known to play patho-physiological role in different body systems including during pregnancy. But its involvement in myometrial spontaneity and associated signalling pathways in uterus in non-pregnant animals is yet to be studied. Present study describes the effect of L-cysteine, an endogenous H_2_S donor, on isolated myometrial strips of non-pregnant buffaloes and the underlying signaling mechanism(s).

**Results:**

L-cysteine (10 nM-30 mM) produced concentration-dependent contractile effect on buffalo myometrium which was extracellular Ca^2+^ and L-type calcium channels-dependent. Significant rightward shift of dose-response curve of L-cysteine was observed with significant decrease in maxima in the presence of amino-oxyacetic acid (AOAA; 100 μM) and d, l-propargylglycine (PAG; 100 μM), the specific blockers of cystathionine β-synthase (CBS) and cystathionine γ-lyase (CSE), respectively. Existence of CBS enzyme of 63 kDa and CSE of 45 kDa molecular weights was confirmed by western blot using specific antibodies and also by immunohistochemistry.

**Conclusions:**

Endogenous H_2_S along with its biosynthetic enzymes (CBS and CSE) is evidently present in uteri of non-pregnant buffaloes and it regulates spontaneity in uteri of non-pregnant buffaloes and this effect is dependent on extracellular Ca^2+^ influx through nifedipine-sensitive L-type calcium channels. Thus H_2_S-signalling pathway may be a potential target to alter the uterine activities in physiology and patho-physiolgical states.

## Background

Hydrogen sulphide (H_2_S), a well known environmental pollutant, has recently been classified as gaso-transmitter along with other members (NO and CO) to regulate several physiological and biological functions. Brain and certain other tissues generate H_2_S from L-cysteine, the endogenous precursor which is catabolised by cystathionine β- synthase (CBS) and cystathionine γ- lyase (CSE). Physiological importance of H_2_S came into existence with the discovery of these constitutive enzymes of H_2_S production and presence of nanomolar concentrations of H_2_S in mammalian blood [1]. Exposure of pregnant rats to L-cysteine on gestational day 19 produced dose-dependent decrease in uterine spontaneous contractility [2]. Recently, increase in expression of CSE (~45 kDa) and CBS (~63 kDa) enzymes in rat and human myometrium during hypoxic condition [3] and dysregulated CSE/H_2_S signalling pathway associated with preeclampsia [4] have been reported. Present study was undertaken to decipher effect of H_2_S on myometrial activity in non-pregnant buffaloes and unravel its mechanistic pathway.

## Methods

### Tissue collection

Uteri along with the ovaries of non-descript buffaloes (*Bubalus bubalis*) were collected from the local abattoir/butcher shop in oxygenated and chilled (4.0 ± 0.5 °C) Ringer Locke solution (RLS) of the composition (mM/L)- NaCl 154, KCl 5.6, CaCl_2_.2H_2_O 2.2, NaHCO_3_ 6.0, d-Glucose 5.5 and having pH of 7.4. Diestrous stage uteri were selected based on the well developed projected (crowned) corpus luteum on ovaries and genitalia with closed cervix and thick mucus [5]. Uteri were also cut open to rule out the possibility of early pregnancy.

### Functional studies

Myometrial strips were prepared as described earlier [6]. Briefly, uterine strips were dissected out from the midcornual region and longitudinal myometrial strips (3 mm × 1 cm) were prepared by carefully removing the endometrial tissues. The uterine strips were mounted in a thermostatically controlled (37.0 ± 0.5 °C) two channels organ bath (Ugo Basile, Italy) of 10 ml capacity containing RLS continuously aerated with carbogen (95% O_2_+ 5% CO_2_) under a resting tension of 2 g. Isometric tension in myometrial strips was recorded with the help of force transducer (Panlab, Spain) using Lab Chart Pro V6.1.3 software (Powerlab, AD Instruments; Australia). During the equilibration period of 2 h, bath fluid was changed after every 10 min. The concentration-dependent (10 nM to 30 mM, at 0.5 log dose unit) response to L-cysteine was recorded in myometrial strips.

Effect of L-cysteine alone or L-cysteine in the presence of different blockers/inhibitors was studied on two different myometrial strips prepared from the uterus of same animal. Each concentration of L-cysteine was allowed to react with the tissue for 8 min to construct the cumulative concentration response curve of L-cysteine alone or in the presence of either L-type Ca^2+^ channels blocker (nifedipine, 100 nM) or H_2_S biosynthesizing enzyme CBS (AOAA, 100 μM) or CSE (PAG, 100 μM) inhibitors. Myometrial strips were incubated with different blockers for 30 min before adding L-cysteine to assess the effect of L-type Ca^2+^-channels blocker or enzyme inhibitors on uterotonic action of L-cysteine.

The time intervals for recording the response of L-cysteine were selected based on the findings of our pilot experiments to record the time taken by the sub-maximal concentration of L-cysteine (3 × 10^−5^) to produce the maximum response in myometrial strips from uteri of non-pregnant buffaloes. A time control experiment was carried out to exclude the possibility of effect of time on myometrial spontaneity during stimulation.

In a separate set of experiment, to assess the role of extracellular Ca^2+^, concentration-dependent response of L-cysteine was recorded in Ca^2+^-free RLS. Removal of Ca^2+^ from RLS resembled with the condition of lack of extracellular Ca^2+^ and thereby the response produced by L-cysteine seemed to be solely dependent on intracellular calcium.

Cumulative concentration–response curves were constructed using the mean integral tension (MIT) data versus concentration [7]. EC_50_ and E_max_/ R_max_ (maximum relaxation) values were determined by non linear regression analysis using Graph Pad Prism 4.0 (GraphPad, La jolla, USA) and pD_2_ value (potency) was calculated as -log of EC_50_ **.** Only one tissue was used from each animal and ‘n’ denotes the number of animals for each tension experiment.

### Western blot studies

Proteins were separated on 10% SDS polyacrylamide gels using GeNEi™ electrophoresis unit and transferred subsequently onto a polyvinylidene difluroide (PVDF) membranes at 10 V for 30 min using GeNEi™ transfer apparatus containing transfer buffer (20 mM Tris, 192 mM glycine and 20% methanol). The membranes were then blocked in a blocking buffer containing 5% (*w*/*v*) skimmed milk powder in PBS (pH 7.4). After washing, the blots were incubated overnight at 4 °C with rabbit polyclonal IgG antibody to CBS (primary antibody) (sc-67,154, Santa Cruz) and rabbit polyclonal IgG antibody to CSE (primary antibody) (sc-135,203, Santa Cruz) diluted (1:200) in PBS containing 0.05% (*v*/v) Tween-20 (PBS-T). The blot was washed and incubated for 1 h at room temperature with goat anti-rabbit IgG conjugated with horseradish peroxidise (Abcam, ab6721) at 1:500 dilution in PBS-T. Immunoreactive protein was detected using DAB system (GeNei™).

### Immunohistochemistry studies

Buffalo myometrial sections were fixed in buffered formalin prior to processing the paraffin sections. Paraffin sections (3 μm) were cut, rehydrated and microwaved in citric acid buffer to retrieve antigens. Sections were incubated with 3% H_2_O_2_ in absolute methanol for 45 min to inhibit endogenous peroxidases, and then with 5% normal goat serum for 45 min to block the unspecific antibody binding. The specific antibodies for CSE (sc-135,203, Santa Cruz) and CBS (sc-67,154, Santa Cruz) were used both in the dilution of 1:200 in humidified chamber at 4 °C and in the control sections, slides were incubated in PBS. Then they were washed 3 times in PBS for 5 min each. Then the slides were treated with goat anti-rabbit IgG and were kept for 30 min, and slides were rinsed thrice in PBS for 5 min each. DAB system was used for immune-labelling and DAB tablets were dissolved in 1 ml of deionised water and working solution prepared was used. Positive reactions gave brown colour in the sections and reaction was terminated before the generalized background staining appeared in the negative control by rinsing with distilled water (DW). Slides were counter stained with Mayer’s haematoxylin for 5 min and rinsed in tap water. DAB stained sections were dehydrated through graded alcohol, cleared in xylene and mounted with DPX.

### Chemicals

L-cysteine, nifedipine, amino-oxyacetic acid or hydroxylamine (AOAA) and d,l-propargylglycine (PAG) were procured from Sigma-Aldrich (USA). Except nifedipine, which was dissolved in ethanol, all other chemicals were dissolved in distilled water. Stock solutions of AOAA and PAG were prepared and stored at -20 °C. Fresh solutions of other chemicals were prepared and further dilutions were made in RLS and used on the same day of preparation. Goat anti-rabbit polyclonal antibody was obtained from Abcam while rabbit polyclonal IgG antibody to CBS (H-300) and rabbit polyclonal IgG antibody to CSE (H-167) were obtained from Santa Cruz.

### Statistical analysis

Normal distribution of data was checked using D’Agostino statistical test and analysis revealed that the data of the present study were normally distributed. Results are expressed as mean ± SEM. Concentration-dependent agonist response data were analyzed by two-way analysis of variance (ANOVA) followed by Bonferroni post-hoc test for determining the statistical differences between L-cysteine alone and in the presence of AOAA (100 μM) or PAG (100 μM) while Student’s ‘t’ test was applied to compare the differences between mean values of phasic versus tonic contractions, E_max_ of L-cysteine alone versus L-cysteine + AOAA or Emax of L-cysteine alone versus L-cysteine + PAG. Difference in values was considered to be statistically significant at *P* < 0.05. All the statistical analysis was performed using GraphPad Prism software.

## Results

### Functional studies

**Fig. 1:**
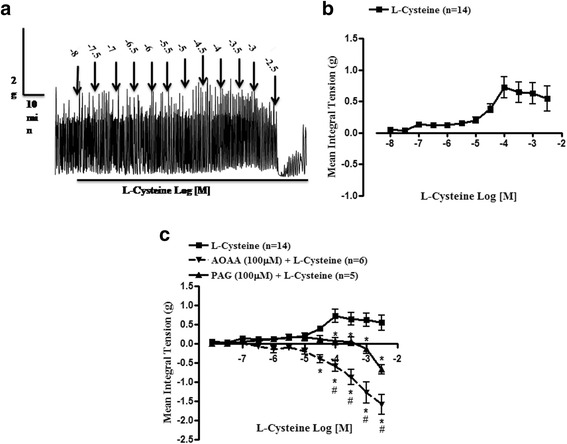
Representative physiograph recording showing the concentration-dependent excitatory effect of L-cysteine (**a**), its cumulative dose response curve (**b**) and comparative cumulative concentration response curves of L-cysteine in the presence of AOAA (100 μM) or PAG (100 μM) on myometrial strips (**c**) from non-pregnant buffaloes. Vertical bars represent SEM. Data were analyzed by two-way ANOVA followed by Bonferroni post-hoc tests. *P < 0.05 L-cysteine vs. AOAA + L-cysteine or PAG + L-cysteine, ^#^P < 0.05 PAG + L-cysteine vs. AOAA + L-cysteine

L-cysteine alone produced concentration-dependent (10 nM to 30 mM, at 0.5 log dose unit) contractile effect on myometrial strips as shown in Fig. 1a and b. The maximum tension (E_max_) and potency (pD_2_) were found to be 0.73 ± 0.17 g and 4.71 ± 0.28, respectively. L-cysteine produced an increase in amplitude up to 100 μM while reduction in amplitude ensued on further addition of L-cysteine. But increase in frequency was observed up to 10 μM. Further, compared to the effect on phasic contraction, L-cysteine significantly (*P* < 0.05) augmented the tonic contractions in buffalo myometrium (Table 1).

**Table 1:** Effect of L-cysteine alone on mean integral tension (E_max_ (g), tonic and phasic contractions (g), amplitude (g), frequency (BPM) and effect on MIT (g) and in the presence of AOAA (100 μM) and PAG (100 μM) in myometrial strips of non-pregnant buffaloes

Myometrial activity markers	Treatment	No. of tissues (n)	pD_2_	E_(max)_ / R _(max)_ (g)
Mean Integral Tension (MIT)	L-cysteine	14	4.70 ± 0.28	0.73 ± 0.17
Phasic contraction	L-Cysteine	6	4.51 ± 0.20	0.11 ± 0.15*
Tonic contraction	L-Cysteine	6	4.63 ± 0.55	0.91 ± 0.22
Amplitude	L-Cysteine	5	7.09 ± 1.75	0.33 ± 0.06
Frequency (BPM) before adding L-cysteine	L-Cysteine	9	……	0.80 ± 0.09
Frequency (BPM) after adding L-cysteine	L-Cysteine	9	3.42 ± 0.83	0.51 ± 0.13
MIT	AOAA (100 μM) + L-Cysteine	6	3.64 ± 0.163	−1.54 ± 0.26^$^
MIT	PAG (100 μM) + L-Cysteine	5	1.37 ± 2.88	−0.67 ± 0.11^$^#

Mean values from two different groups were compared using student’s ‘t’ test

*P < 0.05 Tonic contractions versus phasic contractions

^$^P < 0.05 vs. MIT of L-cysteine, ^#^P < 0.05 vs. MIT of AOAA + L-cysteine

To elucidate the involvement of endogenous biosynthesizing enzymes in H_2_S production, effect of specific enzyme blockers on L-cysteine-induced alterations in myometrial activity was studied. As shown in Fig. 1c, the dose response curve (DRC) of L-cysteine was significantly (P < 0.05) shifted towards right in the presence of AOAA (100 μM) and PAG (100 μM) on buffalo myometrium along with significant decrease in MIT (Table 1). The R_max_ and pD_2_ values of L-cysteine were found to be −1.54 ± 0.26 and 3.64 ± 0.16 in presence of AOAA and −0.67 ± 0.11 and 1.37 ± 2.88 in presence of PAG respectively (Table 1).

**Fig. 2:**
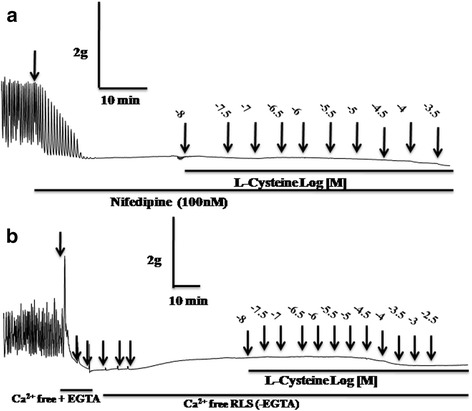
Representative physiograph recording showing the effect of L-cysteine on myometrial strip of non- pregnant buffaloes in the presence of 100 nM nifedipine (**a**; *n* = 6) and in Ca^2+^-free RLS (n = 6) on myometrial strips (**b**) from non- pregnant buffaloes

To elucidate the role of extracellular calcium in L-cysteine-induced uterotonic action in buffaloes, the concentration-dependent (10 nM to 30 mM, at 0.5 log dose unit) response to L-cysteine was recorded either in the presence of nifedipine (a L-type Ca^2+^ channel blocker) or in Ca^2+^-free RLS. As shown in Fig. 2a and b, myometrial spontaneity as well as L-cysteine-induced myometrial contraction was completely abolished in the presence of nifedipine (100 nM) as well as in Ca^2+^-free RLS.

### Western blot studies

**Fig. 3:**
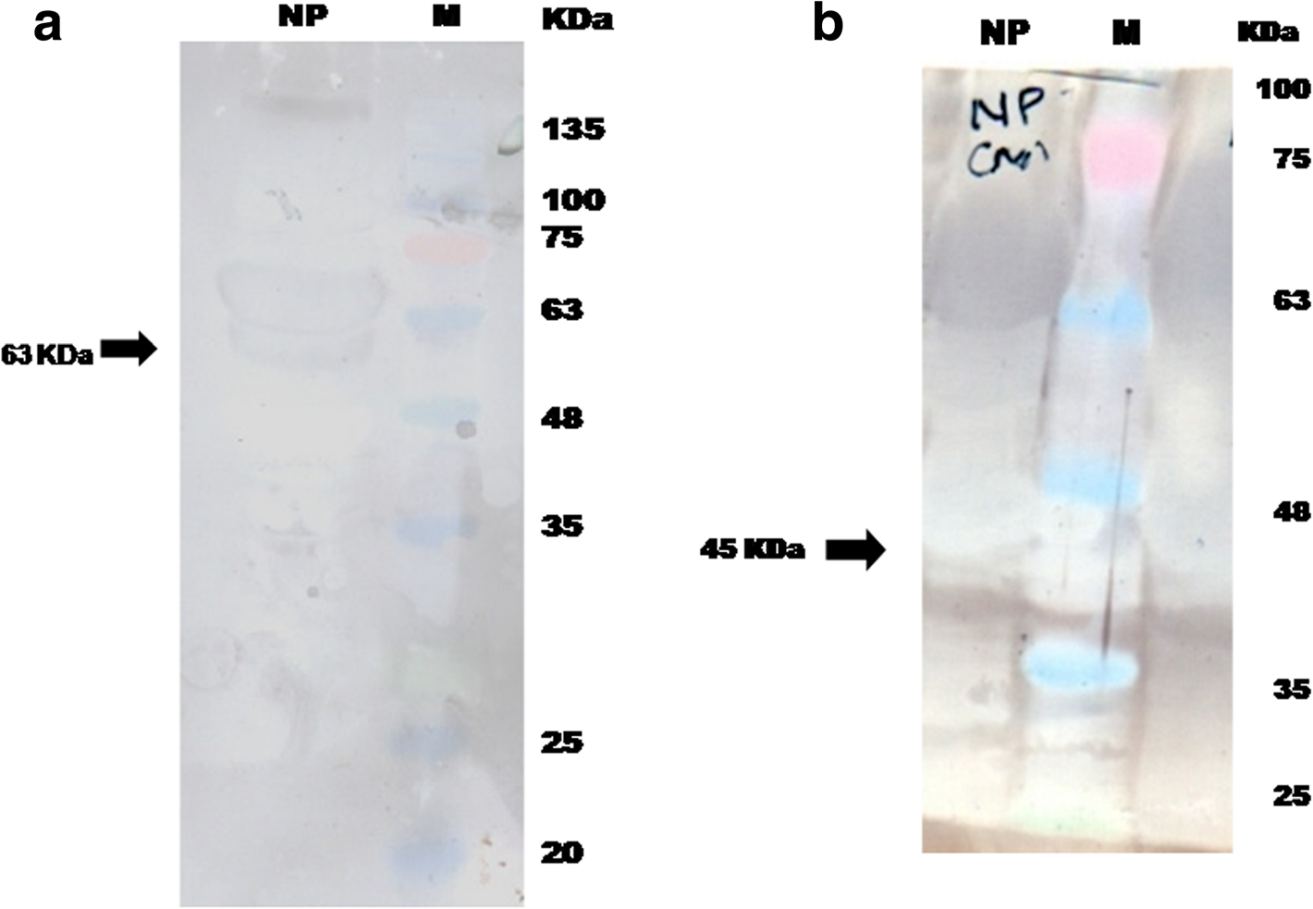
Western blot images showing the specific proteins of approximately 63 kDa of CBS (**a**) and 45 kDa of CSE (**b**) in the proteins isolated from the myometrium of non-pregnant buffaloes. NP - non-pregnant, M – Marker

Western blot analysis of uterine membrane proteins revealed the presence of immune-reactive proteins to CBS (63 kDa) and CSE (45 kDa) enzymes in myometrium of non-pregnant buffaloes (Fig. 3).

### Immunochemistry studies

**Fig. 4:**
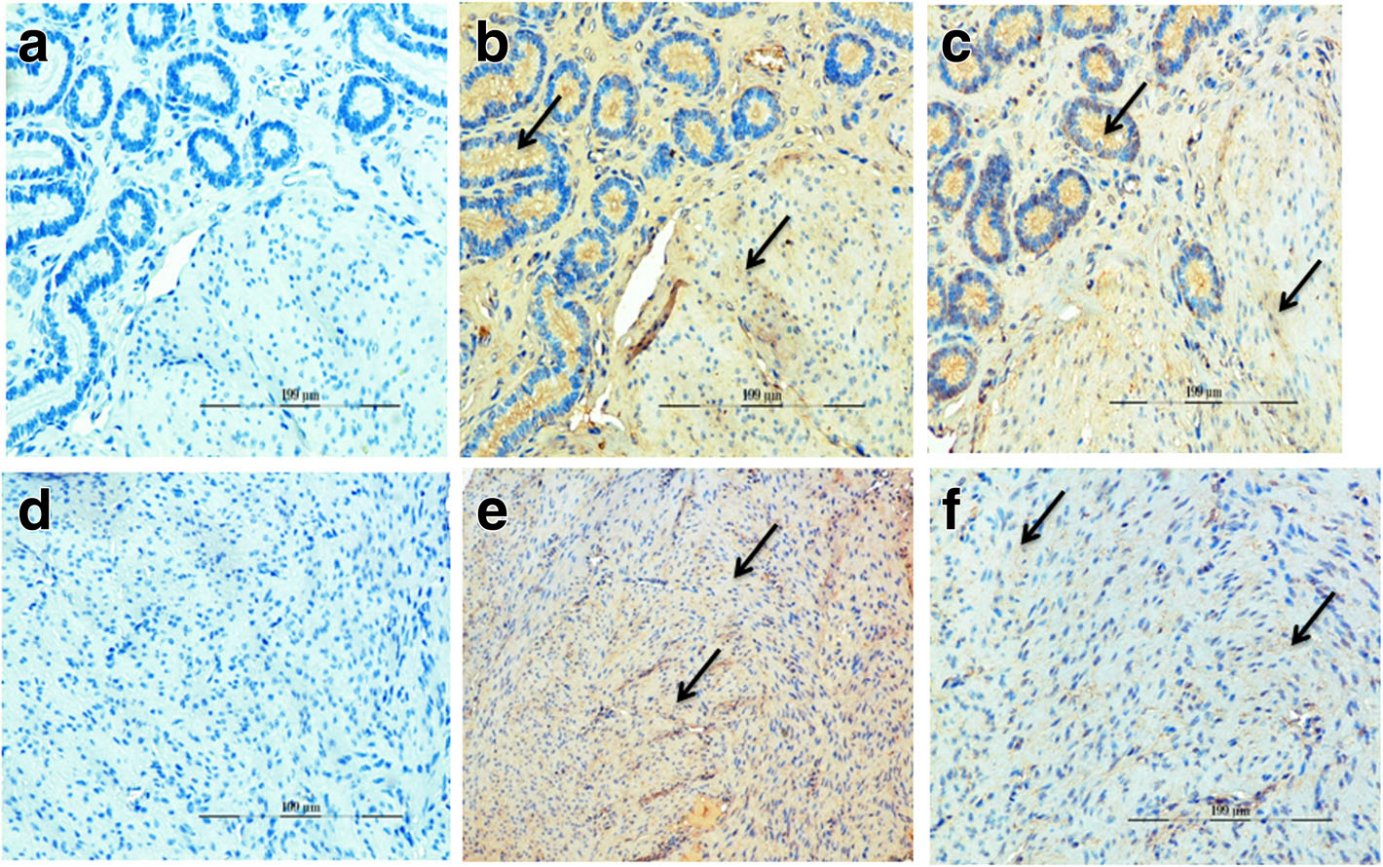
Representative sections of the myometrium of non-pregnant buffaloes showing positive staining for CBS (**b**) and CSE (**c**) in endometrial glands and CBS (**e**) and CSE (f) in smooth muscles of myometrium while **a** and **d** are negative controls. The primary antibody was substituted by normal IgG (**e**) and PBS (**f**). Arrow: positive staining in myometrium smooth muscle and its presence in endometrial glands

Immuno-histochemistry study revealed positive immunoreactivity for CBS as well as CSE enzymes and both these enzymes were predominantly localized in the smooth muscle cells of myometrium (Fig. 4e, f) apart from positive staining of these proteins in the endometrial glands (Fig. 4b, c). Predominant positive signal from CBS (Fig. 4b, e) and weak signal for CSE (Fig. 4c, f) also support the functional involvement of H_2_S in buffalo myometrium (Fig. 1c).

## Discussion

Hydrogen sulphide (H_2_S) is produced either by enzymatic and/or by non-enzymatic process. Among the enzymatic process, L-cysteine is considered to be the main endogenous amino acid substrate which is responsible for production of hydrogen sulphide by the action of two cytosolic enzymes, namely- CBS and CGL/CSE [8–10]. These enzymes were initially discovered in neuronal tissues [11], however, recently their existence has also been reported in rat and human myometrium [3, 12]. L-cysteine is thought to produce its effect on cells through an excitatory amino acid transporter subtype and possibly by a zwitter ion amino acid transporter subtype (EAAT3) [13] and by a zwitter ionic amino acid transporter (ASCT1) [14]. Both these transporters are widely distributed in tissues. Once inside the cell, H_2_S could be released enzymatically from L-cysteine, thus L-cysteine is considered as a direct intracellular donor of H_2_S or HS^−^. Normally L-cysteine is transported into cells as cysteine, which then splits into two molecules of cysteine, but it is possible that L-cysteine could be transported directly when applied extracellularly [15]. On in vitro*-*exposure of isolated myometrial strips of buffaloes to increasing concentrations of L-cysteine (10 nm to 3 mM), concentration dependent uterotonic effect was observed. Rise in frequency of spontaneous contractions as well as tonic contraction by L-cysteine is also reported in non-pregnant rat and in human myometrium [15]. Intracellular concentration of L-cysteine is reported to be 30–200 μM [16] with tissue levels of 10–100 μM [17]. Thus the concentrations used in the present study are well within the range of physiological concentrations of this endogenous amino acid. L-cysteine induced uterotonic action is dependent on activity of CBS and CGL/CSE as evidenced by rightward shift of the DRC of L-cysteine in the presence of enzyme blockers (AOAA or PAG). It was further observed that following blockade of CBS and CGL/CSE enzymes, some relaxant mechanism got activated by L-cysteine to produce uterine relaxation. Therefore, our observations evidently suggest that both these enzymes (CBS and CSE) exist in buffalo myometrium as has been reported in rat and human myometrium [3, 18]. Our assumption about the existence of these bioactivation enzymes in buffalo myometirum based on the functional studies is substantiated by our finding about the existence of CBS and CSE enzymes proteins of the molecular weights of 63 kDa and 45 kDa, respectively, by western blot technique in myometrium of non-pregnant buffaloes as reported in human myometrium [18]. The CBS enzyme was detected at 15 kDa and at 48 kDa in rat and human intrauterine tissues while CSE enzyme was detected at 43 kDa in both rat and human intrauterine tissues [3]. Therefore, our findings evidently suggest about the functional importance of H_2_S in regulating myometrial activity as well as molecular evidence regarding existence of H_2_S producing enzymatic machinery in buffalo myometrium.

Calcium, a universal cellular regulator, is considered to be the primary signal responsible for activation of smooth muscle contractile protein [19, 20]. Cytosolic free calcium, derived from either extracellular source or intracellular pool, phosphorylates myosin light chain to induce cellular contraction. In the present study, role of calcium in L-cysteine-induced myometrial contractility is evident from the fact that following removal of calcium from extracellular fluid (RLS), myometrial spontaneity was completely abolished and this also suggests the role of extracellular calcium in generation and propagation of myometrial spontaneity in buffaloes. Our finding is in agreement with the observations of others where calcium is suggested to play an essential role in uterine myogenic spontaneity both at term [21] and during labour [22]. L-cysteine failed to produce any appreciable contraction in the absence of extracellular calcium and also in the presence of nifedipine, a L-type calcium channel blocker. Therefore, uterotonic action of L-cysteine or L-cysteine-induced myometrial contractility in non-pregnant buffaloes is completely dependent on extra cellular calcium; and nifedipine-sensitive-L-Type calcium channels play the major role as a gateway for entry of extra calcium to mediate uterotonic action of H_2_S. However, role of intracellular calcium cannot be completely ruled out as H_2_S is reported to affect intracellular calcium mobilisation by its action on IP_3_ and ryanodine receptors [23, 24]. Direct inhibitory action of H_2_S on calcium entry through L-type calcium channels is considered to be the underlining mechanism of its relaxant action on pregnant rat myometrium [25] and rat cardiomyocytes [26].

## Conclusions

Our results suggest that L-cysteine-induced contractions in buffalo myometrium is through H_2_S production and it is extracellular Ca^2+^-dependent and both the biosynthesizing enzymes (CBS and CSE) are present in buffalo myometrium and endogenous H_2_S regulates spontaneity in myometrium of non-pregnant buffaloes. Understanding the role of endogenous gaso-transmitter mediators, especially H_2_S, in buffalo myometrium will help in discovery of newer drug targets for addressing the uterine disorders, especially pre-term labour and parturition in buffaloes and other species of animals.

## Abbreviations

AOAA: Aminooxyacetate
CBS: Cystathionine β-synthase
CSE: Cystathionine γ-lyase
H_2_S: Hydrogen sulphide
MIT: Mean integral tension
PAG: D, L-Propargylglycine
PBS: Phosphate buffer saline
RLS: Ringer Locke solution
